# Study of Structure and Mechanical Properties of Fine-Grained Aluminum Alloys Al-0.6wt.%Mg-Zr-Sc with Ratio Zr:Sc = 1.5 Obtained by Cold Drawing

**DOI:** 10.3390/ma12020316

**Published:** 2019-01-21

**Authors:** Aleksey Nokhrin, Iana Shadrina, Vladimir Chuvil’deev, Vladimir Kopylov

**Affiliations:** 1 Lobachevsky State University of Nizhny Novgorod, Nizhny Novgorod 603950, Russia; janashadr@gmail.com (I.S.); chuvildeev@nifti.unn.ru (V.C.); kopylov.ecap@gmail.com (V.K.); 2 Physical-Technical Institute of the National Academy Science of Belarus, Minsk 220141, Belarus

**Keywords:** aluminum alloys, scandium, zirconium, particles, fine-grained structure, hardness, thermal stability

## Abstract

The thermal stability of a fine-grained (FG) aluminum wire has been studied in Al-0.6Mg-Zr-Sc alloys with various scandium and zirconium contents. Specimens were obtained by induction casting followed by cold deformation. The FG alloys have been demonstrated to have high thermal stability of the structure and properties due to the annealing pretreatment (320 °C, 2 h, before drawing), which results in deposition of Al_3_(Sc*_x_*Zr_1−*x*_) intermetallic particles. It has been determined that following a prolonged annealing treatment (400 °C, 100 h), the alloys retain a uniform fine-grained structure with an average grain size of 2.4–2.8 μm whereas their microhardness measures 405–440 MPa.

## 1. Introduction

Fine-grained Al-Sc and Al-Zr aluminum alloys are currently a focus of many research efforts. These alloys have both higher strength and plasticity at room temperature [1,2,3,4,5,6,7,8,9] and exhibit superplasticity at elevated deformation temperatures [10,11,12,13,14,15]. Note that the precipitation of Al_3_(Sc,Zr) particles from an Al-(Sc,Zr) solid solution increases conductivity. This gives grounds to consider fine-grained Al-Sc and Al-Zr alloys as high-strength conductor materials that hold promise for advanced electrical machinery applications. A unique configuration of physical and mechanical properties in fine-grained Al-Sc(Zr) aluminum alloys comes from the positive contributions of Sc, Zr, and Sc + Zr to the mechanical properties of aluminum and Al-based alloys [1,2,3,4,5,7,8,16,17,18,19]. This effect is produced by the capacity of Sc and Zr to form nanodisperse coherent Al_3_Sc and Al_3_Sc(Zr) particles in aluminum alloys that facilitate the formation of a fine-grained structure after recrystallization and to make the recrystallization onset temperature significantly higher [6,10,19,20,21,22], as well as to contribute markedly to higher strengths in aluminum alloys [1,2,3,4,5,8,16,18,19,23,24,25,26,27].

As has been known according to the Orowan equation Δσ_b_ = k_1_Gbf_v_/R^1/2^, the deposition of Al_3_Sc or Al_3_Sc(Zr) increases microhardness H and ultimate strength σ_b_. (Here, R is the particle size, f_v_ is the volume fraction of the particles, G is the shear modulus, b is the Burgers vector, and k_1_ is the numerical coefficient). Many researchers note that the upper limit of increasing the yield strength (Δσ_b(max)_) and microhardness (ΔH_max_) in fine-grained Al-Sc(Zr) aluminum alloys is visibly lower than in coarse-grained alloys of similar compositions [9,28,29,30]. Existing literature [28] shows that the prime cause for that is the rapid growth of size R_z_ in Al_3_Sc(Zr) particles that are deposited on lattice dislocation cores or grain boundaries in aluminum alloys. As has been shown [31], the diffusion activation energy along the grain boundaries and in lattice dislocation cores is approximately two times higher than the activation energy of a lattice diffusion. At the given annealing temperature and time, this considerably expedites particle precipitation and growth and then decreases ΔH_max_ and Δσ_b(max)_. According to the Zener formula, this results in a larger size d_z_ of grains “stabilized” with particles: d_z_ = kR_z_/f_v_ [32] (where k is the numerical coefficient correlated to the particle geometry and f_v_ is the volume fraction of deposited particles). Thus, the maximum increasing yield strength Δσ_b(max)_ in fine-grained aluminum alloys decreases.

A common solution to this problem is to dope fine-grained Al-Sc(Zr) aluminum alloys with magnesium (1.5–6 wt.%), which decreases the grain-boundary diffusion coefficient for aluminum [4,9,10,11,12,13,14,15,20,21,28,33,34,35,36,37,38]. This, in turn, shrinks the deposited Al_3_Sc(Zr) particles, facilitates the formation of a finer-grained structure during annealing, and additionally increases the strength and hardness of Al-Sc(Zr) aluminum alloys [10,21,28,34].

It is important to note that we regard Al-Sc(Zr) alloys as materials for advanced conductor applications. Depending on the specific intended use, this fact entails higher requirements to their strength, plasticity, thermal stability, and conductivity. The high conductivity requirement imposes strong limitations on the maximum concentration of the doping elements in the alloys, limiting the maximum concentrations of Mg, Sc, Zr, and Fe that all drive up electrical resistance linearly even in small concentrations [39]. This has motivated numerous researchers to actively search for doping elements that could effectively substitute for the expensive scandium in the doping of aluminum alloys [3,7,40,41,42,43] and for optimal ratios between Sc and Zr in aluminum alloys [8,18,19,27,44,45,46].

The aim of this study is to investigate the thermal stability of the structure and mechanical properties of new Al-Mg-Sc-Zr aluminum alloys for conductor applications that have a lower Mg content and where the fine-grained structure is stabilized by an alloy ageing pretreatment (before deformation) in its original coarse-grained state.

## 2. Materials and Methods

The study was carried out on Al-0.6 wt.% Mg aluminum alloys with various contents of Sc and Zr (Alloys 1–3). The ratio of Sc and Zr (wt.%) in Alloys 1–3 was Zr:Sc = 1.5. For the chemical composition of the investigated alloys, see Table 1. The chemical composition of the alloys was studied by iCAP 6300-ICP-OES Radial View spectrometer (Thermo Scientific, Waltham, MA, USA).

Alloys were produced using A99 aluminum grade, Mg90 magnesium grade, and master alloys Al-2 wt.% Sc and Al-10 wt.% Zr. The structure of the alloys was studied with a Leica DM IRM interference metallurgical microscope (Leica Microsystems GmbH, Wetzlar, Germany) and Jeol JSM-6490 scanning electron microscope (SEM, Jeol Ltd., Tokyo, Japan) with an Oxford Instruments INCA 350 energy dispersive spectrometer (EDS, Oxford Instruments pls., Oxford, UK). The specimens for the grain-structure study were mechanically polished with diamond suspension and finished to roughness under 1 μm, then etched in an alcohol solution (10.4% HF + 6.3% HNO_3_ + 83.3% C_3_H_8_O_3_). The average size of the grains and particles of the second phase was determined with GoodGrains 2.0 software. 

**Figure 1:**
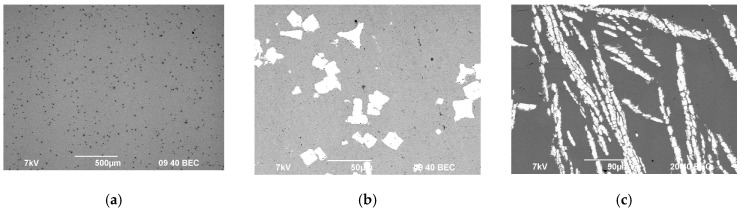
Microstructure of the magnesium master alloy Mg90 (**а**), Al–2Sc (**b**), and Al–10Zr (**c**).

**Figure 2:**
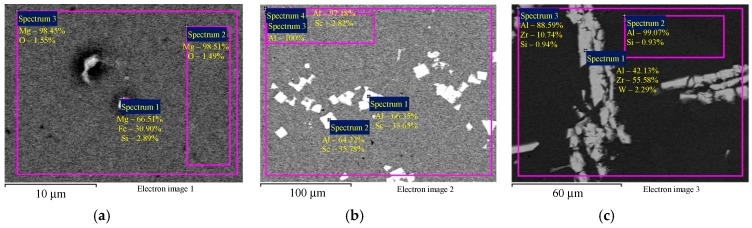
The EDS microanalysis of the composition of the magnesium master alloy Mg90 (**а**), Al–2Sc (**b**), and Al–10Zr (**c**). Numbers (1), (2), and (3), etc. mark the investigated areas corresponding to the markings in the figures. The results are shown in wt.%.

The SEM results with master alloy structures are shown in Figure 1; the EDS results with master alloy compositions are shown in Figure 2. Figure 2 demonstrates that the high Fe content (0.30 wt.%) in the alloys (Table 1) is due to the Fe content in the Mg90 master alloy.

Aluminum alloys of defined compositions (diameter 20 mm specimens) were produced by induction casting in a INDUTHERM VTC-200 vacuum casting machine (Indutherm GmbH, Walzbachtal, Germany) using the following process configurations: the starting melt temperature is 705–710 °C, the Mg introduction temperature is 760 °C, the holding temperature is 850 °C for 3 min, the pouring temperature is 830–850 °C, the cooling rate is over 20 °C/s (copper casting form, zirconia crucible, argon in chamber), and the pyrometer accuracy is ±5 °C.

Conductor-type specimens were in the form of a diameter 0.26 mm wire produced by rolling into a rod to be drawn into a wire at room temperature. Drawing was followed by annealing (320 °C, 2 h) to eliminate internal stress and to facilitate the deposition of the Al_3_(Sc*_x_*Zr_1−*x*_) stabilizing nanoparticles. The wire was produced at the Moscow Special Alloys Processing Plant JSC (Moscow, Russia).

Electrical resistivity (ρ) was measured with an eddy-current SIGMATEST 2.069 instrument (FOERSTER Int., Pittsburgh, PA, USA) with a measurement accuracy ± 0.1 μΩ·cm. Microhardness was measured with a HVS1000 tester (INNOVATEST Europe BV, Maastricht, Netherlands) under load P = 50 g. For this structural state, the result was taken as an average of 20 measurements made on polished surfaces with an average measurement accuracy ±30–35 MPa. The wire microhardness was measured longitudinally and transversely.

Annealing was done in an EKPS-10 forced-air furnace (Smolensk SKTB SPU JSC, Smolensk, Russia) in the range of 200–400 °C. Temperature stability ±5 °C. Specimens were placed in glass containers to minimize oxidation during annealing. The cooldown after annealing was done in open air.

## 3. Results and Discussion

**Figure 3:**
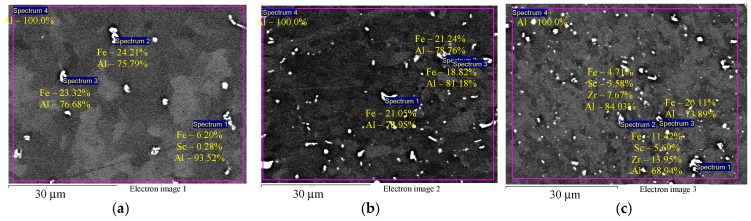
EDS microanalysis of the particle compositions in aluminum Alloys 1 (**а**), 2 (**b**), and 3 (**c**) before drawing and after annealing at 320 °C for 2 h: particles with increased Fe content (**a**,**b**) and particles with increased Zr and Sc content (**c**). The results are shown in wt.%.

Figure 3 shows the microstructure of the investigated alloys in the initial coarse-grained state after annealing at 320 °C for 2 h (before drawing). It can be seen that Alloys 1–3 contain disperse particles comprising Fe, Sc, and Zr. Note that precise determination of the composition by SEM is possible for micron particles only. For submicron particles (below 1 μm), the large beam diameter and excitation area result in a strong unnatural increase of the aluminum concentration in nanoparticles. This artefact precludes the use of the Oxford Instruments INCA 350 EDS analyzer for studying the composition of the nanoparticles that originally precipitate during the annealing of Al-0.6Mg-Zr-Sc alloys. Note also that EDS results presented from Figure 3 onwards are qualitative due to this artifact. The particle size varies within a considerable range from submicron dimensions to several microns. The microhardness of Alloys 1–3 in the initial coarse-grained state after annealing at 320 °C is 580–595 MPa, 650–660 MPa, and 690–710 MPa, respectively.

The measurements of specific electrical resistivity (SER) in the cast specimens after annealing at 320 °C for 2 h (Table 2) show that maximum resistivity values are found in Alloy 2 (ρ_exp_ = 4.0 μΩ·cm) and minimum values are found in magnesium-free Alloy 1 (ρ_exp_ = 3.6μΩ·cm). After drawing, SER increases in all specimens by ~0.05–0.1 μΩ·cm, which is commensurate with the scale of the impact that defects (dislocations and grain boundaries) have on resistivity in pure metals (see [47,48]).

**Figure 4:**
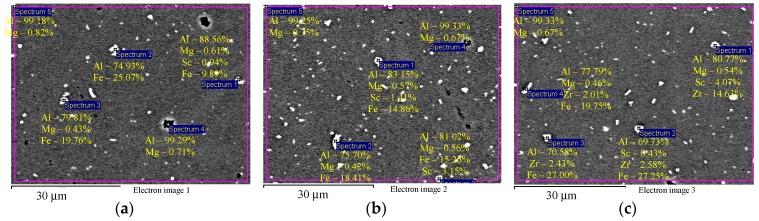
EDS microanalysis of the particle composition in the aluminum alloy specimens after cold deformation: particles with increased Fe content (**а**, **b**) and intermetallic Al_3_(Sc*_x_*Zr_1−*x*_) particles (**c**). The results are shown in wt.%.

EDS microanalysis shows that Alloys 1–3 after cold deformation (drawing) contain particles with an increased Fe concentration (Figure 4а,b) and individual intermetallic Al_3_(Sc*_x_*Zr_1−*x*_) particles (Figure 4c). Average particle size is below 1–2 μm.

As the results indicate, the transverse-section microhardness after drawing (H_v0_) in Alloys 1–3 is 870 ± 35 MPa, 1075 ± 35 MPa, and 1015 ± 20 MPa, respectively. Longitudinal microhardness is 830 ± 55 MPa, 1045 ± 85 MPa, and 1085 ± 65 MPa, respectively. Evidently, cold deformation by drawing has led to an obvious increase in the strength of the alloys.

**Figure 5:**
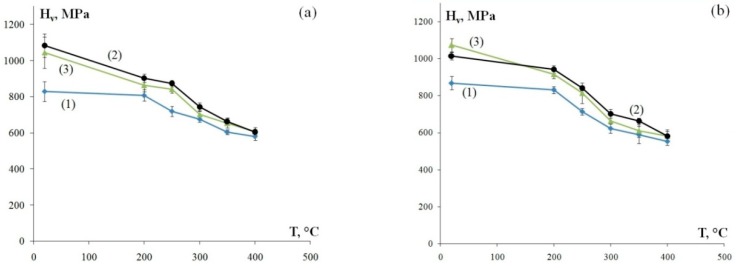
The dependencies of microhardness of the fine-grained aluminum alloys on the temperature of a 30 min annealing treatment: longitudinal section of a wire (**а**) and transverse section of a wire (**b**). The curve numbers correspond to the alloy numbers.

Figure 5 shows the dependencies of microhardness on the temperature of a 30 min annealing treatment. The H_v_(T) dependencies have two stages in all alloys: Stage I of slowly decreasing microhardness during heating up to Т ≤ Т_1_ and Stage II of intensive strength degradation during heating up to Т > T_1_. Note that the value of Т_1_ barely correlates to the chemical composition and stands at ~200 °C (Figure 5). Analyzing the results, it can be seen that alloys with higher original hardness H_v0_ exhibit a more intensive decrease of hardness during Stage I of annealing (Т ≤ Т_1_): in Alloys 2 and 3 with original microhardness H_v0_ = 1015–1085 MPa and 1045–1075 MPa, the average microhardness decrease ΔH_v1_ during heating to Т_1_ = 200 °C is 170 MPa and 130 MPa, respectively; in Alloy 1 with original microhardness H_v0_ = 830–870 MPa and ΔH_v1_ = 30 MPa. A similar microhardness change pattern is seen at Stage II at Т > T_1_: in Alloys 2 and 3, the average microhardness decreases ΔH_v2_ after annealing at 400 °C for 30 min is ΔH_v2_ = 295 MPa and 330 MPa, respectively. The average microhardness change at Stage II of annealing in Alloy 1 (H_v0_ = 830–870 MPa) is ΔH_v2_ = 250 MPa.

**Figure 6:**
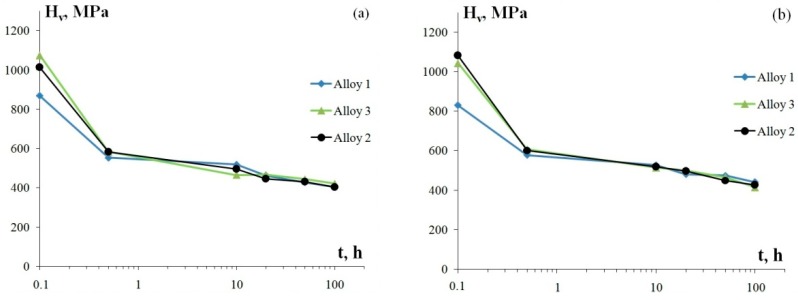
The dependencies of microhardness of the fine-grained aluminum alloys on the time of a 400 °C isothermal annealing treatment: longitudinal section of a wire (**а**) and transverse section of a wire (**b**).

The investigation of the thermal stability of aluminum alloys at 400 °C (100 h) shows that the dependence of microhardness on the isothermal exposure time has two stages in all specimens (Figure 6): a stage of considerably rapid decrease of H_v_ by ~35–45% relative to the original value H_v0_ within the first 30 min of annealing, after which as the exposure time reaches 100 h, the microhardness slightly decreases by ~15–20% in Alloys 1–3. Microhardness in Alloys 1–3 after annealing at 400 °C for 100 h is 405–440 MPa. 

**Figure 7:**
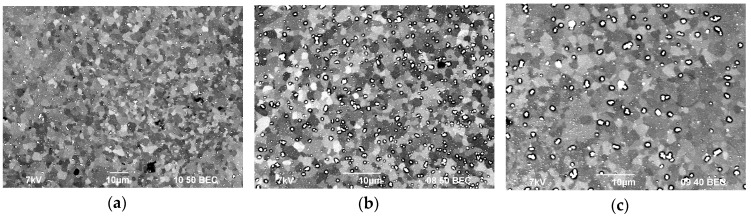
The microstructure of the fine-grained aluminum alloys after annealing at 400 °C for 100 h: Alloy 1 (**а**), Alloy 2 (**b**), and Alloy 3 (**c**).

**Figure 8:**
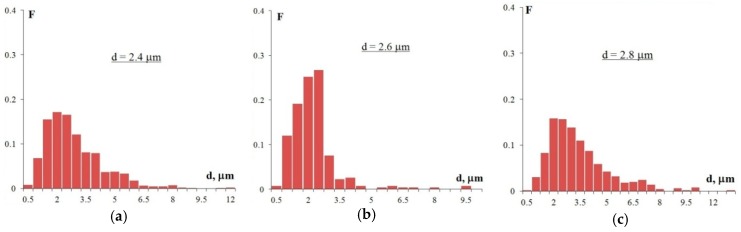
The grain size distribution histograms in Alloy 1 (**а**), Alloy 2 (**b**), and Alloy 3 (**c**) after annealing at 400 °C for 100 h (F is the frequency N(R)/N_Σ_ and d is the grain size).

Figure 7 shows the SEM images of the alloy structures after annealing at 400 °C for 100 h. As the figures show, all alloys have a uniform recrystallized structure. The average grain size, d, in Alloy 1 (Al–0.33Zr–0.25Sc)—d is 2.4 μm, in Alloy 2 (Al–0.20Zr–0.15Sc)—d = 2.6 μm, and in Alloy 3 (Al–0.17Zr–0.11Sc)—d = 2.8 μm. For the grain size distributions, see Figure 8.

**Figure 9:**
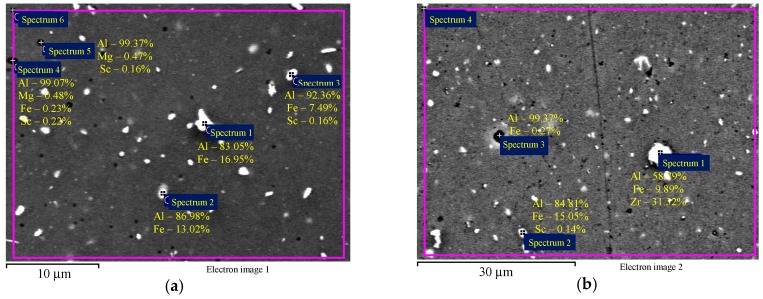
EDS analysis of the particle composition on a polished surface. Annealing at 400 °C for 100 h in Alloy 2 (**а**) and Alloy 3 (**b**). In Figure 9a, Spectrum 6 has 98.88%Al, 0.49%Mg, 0.46%Fe, and 0.17%Sc. In Figure 9b, Spectrum 4 has 99.38%Al, 0.36%Fe, and 0.26%Sc.

The SEM results show that etching of the grain boundaries removes larger particles, the volume percentage of which is the lowest in Alloy 1 and the highest in Alloy 3. Another observation is (see Figure 9) that the alloy structure contains two types of coarse particles: particles (I) with an increased Fe content and particles (II) with an increased content of Sc and Zr that are likely to be Al_3_(Sc*_x_*Zr_1−*x*_) intermetallics [49,50].

**Figure 10:**
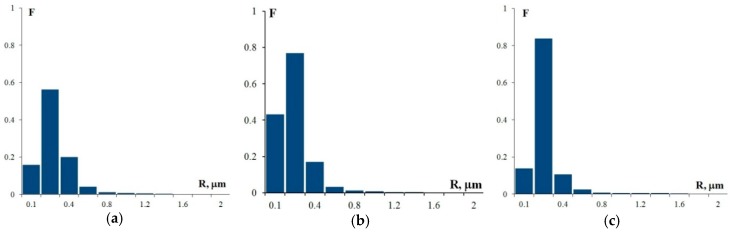
The histograms of the particle size distribution in Alloy 1 (**a**), Alloy 2 (**b**), and Alloy 3 (**c**) after annealing at 400 °C, 100 h (F is the frequency N(R)/N_Σ_ and R is the particle size).

The analysis of the images in Figure 7 reveals that, as the Sc and Zr content in Al–Mg–Sc–Zr alloys decreases, there is a simultaneous decrease in the volume percentage and an increase in the size of the deposited particles. Note that the alloy structure contains two types of particles: coarse particles, the volume percentage of which as measured after annealing at 400 °C for 100 h corresponds with a high accuracy to the size and volume percentage of particles observed in these alloys in their initial coarse-grained state before drawing (see Figure 3), and fine light submicron particles. The average particle size (R_0_) in Alloys 1, 2, and 3 is ~0.28 μm, ~0.15 μm, and ~0.20 μm, respectively. For the particle size distributions, see Figure 10. The mean square deviation for R_0_ depends on the nature of the particle size distribution histogram and stands at 0.1–0.15 μm. 

This leads us to a conclusion that the investigated alloys have a highly stable grain structure due to the deposition of the dispersed Al_3_(Sc*_x_*Zr_1−*x*_) particles.

Now let us analyze the obtained results.

Analyzing the experimental results, in order to calculate the fraction of particles deposited during annealing f_v_(t,Т), we need to determine the maximum resistivity values ρ^max^ at volume fraction zero (f_v_ = 0) and the value ρ_min_ corresponding to a full disintegration of the supersaturated solution (f_v_ = f_v(max)_). To that end, assume that the resistivity contributions of doping elements are adding up [39], and introduce the reference values for resistivity increases caused by respective dopants [39].

Table 2 shows theoretical resistivity values (ρ_th_) of alloys as calculated with the above assumption for Mg, Zr, Fe, and Sc contributing to the resistivity of a solid solution (the Matthiessen’s rule [39]) as well as the experimentally measured resistivity values before the thermal treatments (ρ_exp_). Estimations of the resistivity contributions of Mg, Sc, Zr, and Fe in aluminum were made based on the data in Reference [39]. As can be seen from Table 2, there is an obvious difference between the theoretical and experimental resistivity values ρ_exp_: Δρ = ρ_th_ − ρ_exp_. Assuming that solid solution disintegration only entails the deposition of Al_3_(Sc*_x_*Zr_1−*x*_) intermetallics, we can estimate the maximum possible resistivity change for each Alloy: Δρ_max_ = Δρ_Sc_ + Δρ_Zr_. Note that Δρ_max_ is quite close to Δρ = ρ_th_− ρ_exp_: in Alloy 1 the Δρ_max_ and Δρ values are 1.083 μΩ·cm and 1.179 μΩ·cm respectively and in Alloy 3, Δρ_max_ = 0.525 μΩ·cm and Δρ = 0.521 μΩ·cm, respectively.

We believe this finding indicates that during casting and ageing (annealing at 300 °C for 2 h) before drawing, almost a complete disintegration of the supersaturated Sc and Zr solid solution took place in Al followed by the deposition of intermetallic Al_3_(Sc*_x_*Zr_1−*x*_) nanoparticles. This outcome is in agreement with the data in References [28,51] on the kinetics of the disintegration of the Sc and Zr solid solution in Al-Mg-0.22Sc-0.15Zr alloys with different Mg contents (0, 1.5, 3, 4.5 wt.%) where it has been shown that Al-0.22Sc-0.15Zr and Al-1.5Mg-0.22Sc-0.15Zr exhibit intensive deposition of Al_3_(Sc*_x_*Zr_1−*x*_) particles after heating to 240 °C and that after annealing at 300 °C for 2 h a ~50% disintegration of the solid solution takes place alongside with a ~0.4 vol.% deposition of Al_3_(Sc*_x_*Zr_1−*x*_) particles.

The procedure detailed in [51] and applied to calculate the maximum volume fraction (f_v0_) of the deposited intermetallic particles shows that in Alloys 1–3, the values of f_v0_ are ~1.0 vol.%, ~0.6 vol.%, and ~0.48 vol.%, respectively. The calculations rest on the assumption that annealing only entails the deposition of Al_3_(Sc*_x_*Zr_1-x_) particles and that the solubility limit for Sc and Zr in Al at 300 °C is zero (see References [1,52,53]). Calculating the stable grain size with the Zener formula d_z_ = 3/4·R_z_/f_v0_ [32], the average size of the intermetallic Al_3_(Sc*_x_*Zr_1−*x*_) particles required to stabilize the grain structure in Alloys 1–3 is R_z_ = 180 nm, 325 nm, and 435 nm, respectively. The calculated grain sized R_z_ for intermetallic particles are well aligned with the published experimental data [13,16,17,18,19,23,24,26,28,34], and measurements of the average size of R_0_ particles. The difference between the theoretical value R_z_ and the experimentally observed value R_0_ is apparently caused by the fact that experimental R_0_ is taken as an averaged size of Al_3_(Sc,Zr) and Al–Fe particles.

Generalizing the experimental outcomes and their analysis, we can conclude that annealing pretreatment (before deformation) of cast aluminum alloys is an effective technique to stabilize their fine-grained structure given it is followed by severe plastic deformation and high-temperature thermal treatment.

In closing, below is a brief discussion of the role that annealing pretreatment (320 °C, 2 h) plays in ensuring thermal stability of the fine-grained structure in Al–0.6Mg–Zr–Sc aluminum alloys. As has been demonstrated above, annealing pretreatment (320 °C, 2 h) of coarse-grained Al–0.6Mg–Zr–Sc alloys results in deposition of Al_3_(Sc*_x_*Zr_1−*x*_) particles as can be seen from the resistivity measurements. It is important to note that during the annealing of coarse-grained alloys, the deposition of Al_3_(Sc*_x_*Zr_1−*x*_) particles occurs within the lattice [51], unlike the annealing of fine-grained alloys where the particle deposition occurs mostly along the grain boundaries or in lattice dislocation cores. As is known, at lower temperatures, below 0.5–0.6T_m_, the diffusion coefficient for lattice diffusion (D_v_) is several orders of magnitude lower that the diffusion coefficient for diffusion on non-equilibrium grain boundaries (D_b_) and lattice dislocation core diffusion (D_c_) in fine-grained alloys obtained by severe plastic deformation. The dependence of the volume fraction of the second-phase particles on the time (t) and temperature (T) of annealing can be described with the Johnson–Mehl–Avrami–Kolmogorov equation: f_v_(t,T) = f_v0_(1−exp(−(t/τ)^n^)), where f_v0_ is the maximal volume fraction of the second-phase particles that precipitate from the solid solution at a given Т, τ = τ_0_exp(Q/kT) is the regular process duration, Q and n are the activation energy and numerical coefficient (disintegration intensity coefficient) to describe the disintegration mechanism [51,54]. The dependence of second-phase particle size (R) on the time (t) and temperature (T) of annealing can be described with an equation [32]: R^m^ − R_0_^m^ = ξ_1_Dt, where R_0_ is the initial particle size, m is the numerical coefficient to describe the particles growth mechanism [54], ξ_1_ is the numerical coefficient dependent on the geometric and thermodynamic properties of the material [54], D = D_0_exp(−Q/kT) is the diffusion coefficient, and D_0_ is the pre-exponential multiplying factor. The consequence of this is that the rates of deposition and growth of Al_3_(Sc*_x_*Zr_1−*x*_) particles in fine-grained alloys is considerably higher than these rates in coarse-grained aluminum alloys. We believe that this prevents researchers from attaining the desired increase in thermal stability of the fine-grained structure in highly deformed alloys since, according to the Zener formula, intensive particle growth would lead to a proportional increase of average grain size in the alloy. In light of this, the annealing pretreatment (before severe plastic deformation), which ensures almost complete precipitation of the particles within the lattice and, consequentially, slows down the rate of their growth during further annealing treatments (after severe plastic deformation), ensures better stability of the fine-grained structure in Al–Mg–Sc–Zr aluminum alloys.

In the analysis of the dependence of microhardness on the annealing temperature, as is shown in Figure 5, the intensity of the microhardness change during Stage I (Т ≤ Т_1_) varies across fine-grained alloys of different compositions. Note that as the total content of zirconium and scandium in the alloy (Zr + Sc) goes higher, the magnitude of microhardness decrease during Stage I of annealing goes lower. As can be seen in Figure 5, the lowest microhardness in the initial state (H_v0_) and the lowest value of microhardness during Stage I of annealing (ΔH_v1_) is observed in fine-grained Alloy 1 with the highest content of scandium and zirconium (Zr + Sc = 0.77 wt.%) and the reverse is observed in Alloy 3 with the lowest content of Zr + Sc = 0.28 wt.% (see Table 1). This result is rather unexpected. The conventional assumption is that higher concentrations of Sc and Zr lead to higher microhardness in aluminum alloys (e.g., see References [1,2,3,7,16,18,19]).

Note that the alloys were produced under identical casting conditions regardless of Sc and Zr concentration. Consequentially, the amounts of primary Al_3_(Sc,Zr) particles formed during casting would be markedly higher in the alloys with higher Sc and Zr content (Alloy 1) as compared to Alloy 3 where the total concentration of Sc and Zr (Sc + Zr = 0.28 wt.%) is below the solubility limit of Sc and Zr in aluminum. The total concentration of Sc and Zr in Alloy 2 (Sc + Zr = 0.35 wt.%) is slightly higher than the total concentration of Sc and Zr that can be “dissolved” in the aluminum lattice (~0.32 wt.% [52]). It can, therefore, be expected that increasing the total concentration of Sc and Zr will lead to increasing the volume fraction of relatively large (submicron and micron) primary particles that precipitate during melt crystallization.

As has been demonstrated above, during annealing at 320 °C for 2 h, the deposition of Al_3_(Sc,Zr) nanoparticles occurs within the aluminum lattice that then grow slowly during further annealing of fine-grained aluminum.

Due to this circumstance, the two-staged nature of microhardness dependence may be linked to the presence of two types of particles in the Al–0.6Mg–Zr–Sc structure that precipitate during the production of aluminum studs (casting followed by annealing at 320 °C for 2 h). These are larger primary submicron particles that can be observed in SEM (see Figure 3 and Figure 4) and secondary nanodisperse particles that depose mostly after annealing at 320 °C for 2 h. The low value of initial microhardness H_v0_ and the small magnitude of microhardness change during Stage I of annealing ΔH_v1_ are caused, we reckon, by all Zr and Sc atoms being retained by primary submicron particles during casting (crystallization of Alloy 1) and, consequentially, low amounts (volume fraction) of secondary nanoparticles being deposited during annealing at 320 °C for 2 h. During the casting of Alloy 1, this prevents higher concentrations of Zr and Sc in the alloy lattice and prevents the increasing initial microhardness values H_v0_ in Alloy 1 up to 1015–1085 MPa, which would correspond to the initial microhardness of Alloys 2 and 3 where Sc and Zr concentrations are lower than in Alloy 1 but still close to the solubility limit of Sc and Zr in aluminum.

**Figure 11:**
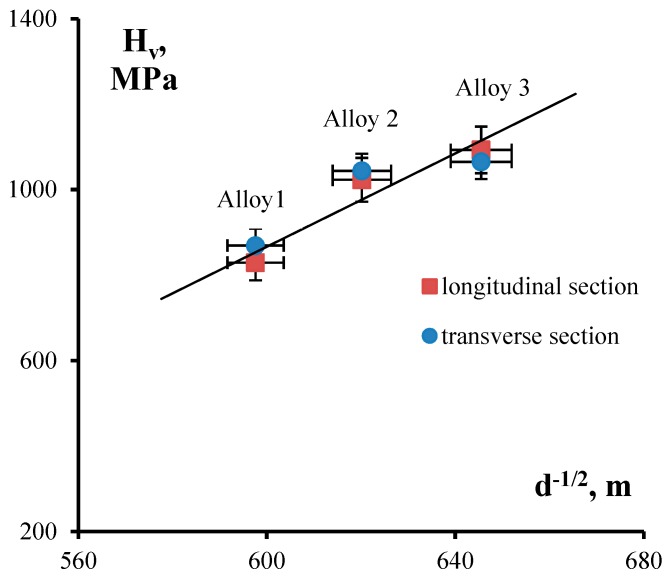
Dependence of microhardness on grain size as expressed in coordinates H–d^−1/2^. Analysis of experimental data from alloys annealed at 400°C for 100 h.

During Stage I of annealing, nanodispersed Al_3_(Sc*_x_*Zr_1−*x*_) particles grow to submicron sizes and, according to the Orowan equation, there is a decrease of microhardness in fine-grained Alloys 2 and 3. According to the Zener formula, the growth of Al_3_(Sc*_x_*Zr_1−*x*_) nanoparticles results in increasing average grain size up to its stable size of 2.4–2.8 μm, which we believe to be determined by the presence of primary Al_3_(Sc*_x_*Zr_1−*x*_) submicron particles in the alloy structure. Grain growth during Stage II of annealing leads to the lowering of alloy microhardness in line with the Hall–Petch equation: ΔH_v_ − A/d^1/2^, where A is the coefficient of grain-boundary strengthening. This outcome is also indirectly corroborated by the fact that the conditions of the Hall–Petch relationship are satisfied in alloys annealed at 400 °C for 100 h (see Figure 11).

## 4. Conclusions

New fine-grained aluminum alloys with lower magnesium content were designed, suitable for conductor applications and possessing high thermal stability: the average grain size in a wire made of the new alloys is 2.4–2.8 μm after annealing at 400 °C for 100 h and the microhardness is 405–440 MPa. Annealing pretreatment (before drawing) at 320 °C for 2 h, which results in the deposition of intermetallic Al_3_(Sc*_x_*Zr_1−*x*_) nanoparticles and a virtually complete disintegration of Sc and Zr solid solution in Al, has been determined to produce higher thermal stability of a fine-grained structure in intensively deformed aluminum alloys.

High thermal stability of the fine-grained structure in Al–0.6Mg–Zr–Sc aluminum alloys is caused by the slow rate of growth of Al_3_(Sc*_x_*Zr_1−*x*_) particles that precipitate within the lattice during the annealing pretreatment (320 °C for 2 h).

## Figures and Tables

**Table 1:** Chemical composition of aluminum alloys.

Element	Average Content, wt.%		
	Alloy 1	Alloy 2	Alloy 3
Si	0.030	<0.010	0.010
Fe	0.30	0.40	0.30
Cu	0.003	0.003	0.006
Mn	0.004	0.005	0.003
Mg	0.60	0.60	0.60
Zn	0.001	<0.001	<0.001
Ga	<0.001	<0.001	<0.001
Ti	<0.001	<0.001	<0.001
As	<0.001	<0.001	<0.001
Zr	0.33	0.20	0.17
Sc	0.25	0.15	0.11
Al	Base	Base	Base

**Table 2:** Experimental and theoretical specific electrical resistivity of aluminum alloys.

Alloys	Contribution of					Theory ρ_th_, μΩ·cm	Experiment ρ_exp_, μΩ·cm	Δρ = ρ_th_−ρ_exp_, μΩ·cm
	Al ρ_Al_, μΩ·cm	Mg Δρ_Mg_, μΩ·cm	Fe Δρ_Fe_, μΩ·cm	Zr Δρ_Zr_, μΩ·cm	Sc Δρ_Sc_, μΩ·cm			
No. 1	2.655	0.328	0.713	0.585	0.498	4.779	3.6	1.179
No. 2	2.655	0.328	0.903	0.351	0.299	4.536	4.0	0.536
No. 3	2.655	0.328	0.713	0.293	0.232	4.221	3.7	0.521
